# De Novo Designed Minibinders Targeting the GDF15–GFRAL Axis Reverse Cancer Cachexia and Restore Anti‐Tumor Immunity

**DOI:** 10.1002/advs.76202

**Published:** 2026-07-07

**Authors:** Haitao Wang, Tianzhen Hua, Meiling Wang, Bingbing Meng, Yue Zhang, Chengxu Jiang, Yunhe Gao, Hongqi Yang, Jian Bo

**Affiliations:** ^1^ Senior Department of Hematology The Fifth Medical Center of Chinese PLA General Hospital Beijing China; ^2^ Chinese PLA Medical School Beijing China; ^3^ Naval Medical University Shanghai China; ^4^ University of Montpellier Montpellier France; ^5^ Senior Department of General Surgery The First Medical Center of Chinese PLA General Hospital Beijing China; ^6^ Department of Emergency The Second Medical Center of Chinese PLA General Hospital Beijing China

**Keywords:** cancer cachexia, de novo protein design, GDF15, immune checkpoint blockade, RFdiffusion

## Abstract

Cancer‐associated cachexia is a devastating syndrome characterized by progressive weight loss, reduced survival, and impaired responses to anticancer therapies. Growth differentiation factor 15 (GDF15), acting through its receptor GFRAL, has emerged as a key mediator of cachexia, yet effective and mechanistically defined strategies to neutralize this pathway remain limited. Here, we applied structure‐guided de novo protein design to generate compact minibinders that selectively target the GDF15–GFRAL interaction interface. Using an integrated computational pipeline combining RFdiffusion, ProteinMPNN, and AlphaFold 3 structure prediction, we designed and experimentally validated high‐affinity GDF15 minibinders with picomolar‐range binding affinities and exceptional structural stability. Mutagenesis and charge‐complementary rescue experiments confirm that these minibinders neutralize GDF15 through precisely engineered interface contacts. Functionally, the minibinders suppress GDF15–GFRAL signaling, inhibit downstream transcriptional responses, and robustly reverse cachexia in vivo across multiple tumor models, resulting in significant improvements in body weight and survival. Importantly, neutralization of GDF15 also restores sensitivity to anti–PD‐1 immunotherapy in a GDF15‐driven resistant tumor model. Combination treatment enhances CD8^+^ T cell infiltration and effector function within tumors, and its antitumor efficacy is strictly dependent on CD8^+^ T cells. Together, these findings demonstrate that de novo designed GDF15 minibinders can achieve potent, mechanism‐defined neutralization of the GDF15–GFRAL axis in vivo, translating into robust physiological benefits and restoration of immunotherapy efficacy.

## Graphical abstract

Tumor‐derived GDF15 drives cancer cachexia and suppresses antitumor immunity. Here, de novo designed minibinders targeting the GDF15–GFRAL interface achieve picomolar affinity and potent pathway blockade. The minibinders alleviate cachexia, restore CD8^+^ T‐cell infiltration, enhance anti–PD‐1 responsiveness, and provide survival benefits across tumor models, highlighting a compact therapeutic modality for GDF15‐driven disease.

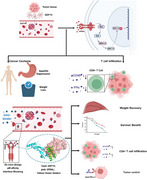

## 1 Introduction

Growth differentiation factor 15 (GDF15) is a stress‐responsive cytokine belonging to the transforming growth factor‐β (TGF‐β) superfamily that is robustly induced under conditions of cellular stress, tissue damage, and diseases [1, 2, 3, 4]. Circulating GDF15 levels are elevated in a wide range of pathological contexts, including cancer, cardiovascular disease, chronic inflammation, infection, and metabolic disorders, and consistently correlate with disease severity and adverse clinical outcomes [5, 6, 7]. These observations have positioned GDF15 as both a biomarker of systemic stress and an active endocrine regulator of organismal physiology.

The physiological actions of GDF15 are mediated through its cognate receptor GFRAL, which is selectively expressed in the area postrema and nucleus tractus solitarius of the brainstem [2]. Engagement of the GDF15–GFRAL axis elicits potent effects on appetite regulation, energy balance, and aversive signaling, enabling peripheral stress signals to influence central nervous system–controlled behaviors [8, 9, 10, 11]. In the context of cancer, sustained elevation of GDF15 contributes to anorexia, weight loss, and metabolic dysregulation, hallmark features of cancer‐associated cachexia [9, 12]. Beyond metabolic control, emerging evidence suggests that GDF15 also modulates antitumor immunity and therapeutic responsiveness, implicating this pathway in shaping both systemic physiology and treatment outcomes [13, 14].

Given its broad involvement across disease states, GDF15 has attracted significant interest as a therapeutic target. Several therapeutic strategies targeting the GDF15–GFRAL signaling axis have recently been explored, primarily focusing on monoclonal antibodies directed against either GDF15 or its receptor GFRAL, as well as GDF15 analogues [12, 15]. These approaches have demonstrated promising preclinical and early–stage clinical potential in cancer cachexia and metabolic disorders. In addition to antibody‐based modalities, alternative approaches such as peptide antagonists targeting GDF15 signaling have also been reported [16]. Nevertheless, compact de novo designed protein binders that directly and mechanistically target the GDF15–GFRAL interaction interface remain largely unexplored. However, existing strategies rely almost exclusively on monoclonal antibodies, which are inherently constrained by large molecular size, limited tissue penetration, and restricted flexibility in targeting specific protein–protein interaction interfaces. Moreover, antibodies are typically optimized through empirical screening rather than explicit, structure‐guided engineering of the GDF15–GFRAL interaction surface. These limitations underscore the need for alternative therapeutic modalities capable of precisely and mechanistically targeting GDF15 signaling.

Recent advances in computational protein design have enabled the de novo generation of compact binding proteins that engage defined molecular surfaces with high affinity and specificity [17, 18, 19]. Methods such as RFdiffusion, ProteinMPNN, and AlphaFold‐based structure prediction now allow rational design of proteins that directly target protein–protein interaction interfaces with atomic‐level precision [20, 21, 22]. De novo designed minibinders offer several potential advantages over antibodies, including reduced size, enhanced stability, and the ability to encode specific mechanistic intent into the binding interface [18, 23]. These properties make minibinders particularly well suited for targeting endocrine ligands such as GDF15, where precise blockade of receptor engagement is required.

In this study, we apply structure‐guided de novo protein design to generate first‐in‐class minibinders that directly target the GDF15–GFRAL interaction interface. We demonstrate that these designed binders achieve potent and mechanism‐ based neutralization of GDF15 signaling, translate into robust physiological benefits in vivo, and exhibit strong translational potential as a new modality for modulating GDF15‐driven disease processes.

## 2 Materials and Methods

### 2.1 Two Complementary Scaffold Design Strategies for GDF15 Binders

To generate antagonistic binders targeting the GDF15‐GFRAL interaction site, we adopted a de novo design approach based on diffusion models [20, 24]. First, we performed an interaction analysis of the GDF15‐GFRAL complex (PDB: 5VZ4) [25], and based on this analysis, we identified key hotspot residues on GDF15 (S35, V87, V96) as constraints to guide the formation of the binding interface. We generated the binder backbone using RFdiffusion. Designs containing more than three secondary structure elements were retained to enrich for compact and stable folds. The scaffold length was limited to 60‐80 amino acids. To increase scaffold diversity, we adjusted the parameters noise_scale_ca (0 or 0.5) and noise_scale_frame (0 or 0.5), which control the perturbation of the backbone Cα coordinates and the local reference frame of each residue. This allows us to introduce controlled flexibility in the local conformation while preserving the overall fold topology, enabling the fine‐tuning and diversification of the interface region. After generating the scaffold, ProteinMPNN was used to design 30 sequences for each scaffold, and the monomer quality of the binders was assessed using ESMFold (with pLDDT > 80). After screening with ESMFold [26], the sequences were further evaluated using AlphaFold3, with selection criteria including pLDDT > 80, pAE_interaction < 10, ΔΔG ≤ −30 kcal/mol, and favorable interface geometry. The candidates that met these criteria were selected for yeast display validation.

In addition, we adopted a second strategy. The BindCraft design process was employed [27], with the GDF15 three‐dimensional structure (PDB: 5VZ4) as input. The design parameters were specified via a JSON configuration file, including the PDB file of GDF15 and its relevant chains, the hotspot residues on GDF15 (such as S35, V87, and V96), the binder length (60‐80 amino acids), and the target number of effective designs (2000). The final candidates were filtered based on several structural and energy metrics, including the overall and per‐residue pLDDT predicted by AF2 Monomer, the interface consistency in the AF2 Multimer complex, and Rosetta‐calculated binding energy, interface energy, and structural clash scores [28]. Designs that met all the screening standards were considered to have high structural reliability and potential binding ability and were selected for subsequent experimental validation.

### 2.2 Expression and Purification of GDF15 Binder and Recombinant Proteins

Expression and Purification of GDF15 Binders, GDF15, and GFRAL Genes encoding the computationally designed GDF15 binders were synthesized and cloned into the pET21b vector. The resulting GDF15 binder–His_6_ recombinant plasmids were transformed into Escherichia coli BL21 (DE3) cells and cultured in LB medium supplemented with 100 µg/mL ampicillin until the cultures reached an OD_600_ of 0.6–0.8. Protein expression was induced with 0.4 m _m_ IPTG, followed by incubation at 20°C for 16 h. Cells were harvested by centrifugation and resuspended in lysis buffer containing 50 mm Tris–HCl (pH 8.0), 150 mm NaCl, and 30 mm imidazole, then lysed by sonication on ice. Cell debris was removed by centrifugation at 15 000 × g for 30 min, and the clarified supernatant was loaded onto Ni‐NTA agarose resin (Sangon Biotech) for His‐tag affinity purification. Bound proteins were eluted using lysis buffer supplemented with 500 mm imidazole. The eluates were further purified by size‐exclusion chromatography (SEC) on a Superdex 75 Increase 10/300 GL column (#GE29‐1487‐21, Cytiva). Purified proteins were concentrated to 1 mg/mL, aliquoted, and stored at −80°C. For mammalian expression, genes encoding GDF15 and GFRAL were cloned into mammalian expression vectors. The GDF15 construct contained an N‐terminal FLAG tag followed by a His_6_ tag, whereas the GFRAL construct contained a His_6_ tag; in both cases, a thrombin protease cleavage site was introduced between the tag(s) and the target protein. Recombinant plasmids were transfected into Expi293F cells (#A14527, Thermo Fisher Scientific; 2 × 10^6^ cells/mL) using polyethylenimine (PEI) (#24765‐2, Polysciences). Cells were cultured at 37°C under 8% CO_2_ with shaking for 5 days, and expression enhancers were added 18–22 h post‐transfection. At the end of the expression period, culture supernatants were collected and clarified by centrifugation to obtain soluble secreted proteins for purification. Clarified supernatants were subjected to His‐tag affinity purification using Ni‐NTA agarose resin (C600791, Sangon Biotech). The resin was pre‐equilibrated with binding/wash buffer (20 mm Tris–HCl, pH 8.0, 150 mm NaCl, 30 mm imidazole), followed by loading of the supernatant and incubation to allow binding. After washing with buffer containing 30 mm imidazole to remove nonspecifically bound proteins, target proteins were eluted with 500 mm imidazole. Ni‐NTA eluates were buffer‐exchanged to conditions suitable for enzymatic cleavage, after which thrombin was added to remove the affinity tags. The digestion was carried out overnight at 4°C under gentle conditions. The reaction mixtures were subsequently passed through Ni‐NTA resin again to remove residual His‐tagged fragments and uncleaved protein. Fractions corresponding to the main SEC peak were collected, concentrated to 5 mg/mL using ultrafiltration centrifugal filters, aliquoted, and stored at −80°C.

### 2.3 Yeast Surface Display

Genes encoding the top 1000 binder candidates were synthesized (Twist Bioscience) and cloned into the yeast display vector pETCON, followed by transformation into Saccharomyces cerevisiae EBY100 using the lithium acetate/PEG method. Transformants were selected on SD–Ura–Trp (SD‐UT) plates at 30°C for 48 h to generate the initial library [29]. For selection, yeast libraries were expanded in SD‐UT, induced in galactose‐containing SG‐UT medium for surface expression, and incubated with FLAG‐tagged GDF15 (1 µm) at 4°C for 1 h. Cells were labeled with anti‐HA and anti‐FLAG antibodies to assess display and target binding, respectively. Fluorescence‐activated cell sorting (FACS) was performed on a BD FACSAria II, gating for HA/FLAG double‐positive cells. Approximately 1 × 10^5^ cells were collected per round, expanded, and subjected to iterative cycles of induction and selection. After 3–4 rounds, enriched clones were isolated, sequenced, and selected for downstream expression, purification, and affinity characterization.

### 2.4 SPR Measurement of Binding Kinetics

Binding kinetics were measured using the Biacore T200 system equipped with CM5 Series S sensor chips. Recombinant GDF15 dimers were immobilized onto the sensor chip surface via amine coupling, using 10 mm sodium acetate (pH 5.5) as the coupling buffer. The immobilization level was controlled to remain in a low RU range to minimize mass transport limitations.HBS‐EP+ buffer was used as both the running and sample buffer. The analytes, including GDF15 binders 05_41_14 and 05_25_1, were diluted in HBS‐EP+ and injected over the immobilized GDF15 sensor chips at concentrations ranging from 3.125 to 50 nm (3.125, 6.25, 12.5, 25, and 50 nm). The injection was conducted at a flow rate of 35 µL/min for 350 s association, followed by a 350 s dissociation phase. After each cycle, the chip surface was regenerated with 10 mm glycine (pH 2.0). Association and dissociation curves were fitted using a 1:1 Langmuir binding model to determine the k_on and k_off rate constants, from which the equilibrium dissociation constant K_D was calculated (K_D = k_off/k_on). All data analysis was performed using Biacore Insight Evaluation Software.

### 2.5 Site‐Saturation Mutagenesis of GDF15 Antagonistic Binders

The site‐saturation mutagenesis (SSM) approach was used to study the interface residues of binder proteins and their binding interactions with GDF15 [30]. First, a library of site‐saturated mutants was generated by introducing mutations at selected interface residues of GDF15 and the binder proteins using degenerate codons. The library underwent two rounds of affinity‐independent selection to remove non‐functional clones, followed by a third round of affinity‐based selection, using gradually decreasing concentrations of 05_41_14 and 05_25_1 (from 5 nm to sub‐nanomolar concentrations). After each round, the selected population was analyzed by deep sequencing using the Illumina NextSeq platform. The sequencing data were assembled and analyzed using PEAR software, from which ΔlogKD values for each mutation were derived, and heatmaps were generated to visualize the effects of individual mutations on binding affinity. Based on the results from the SSM heatmap, rescue mutations were introduced to restore binding affinity, and SPR and luciferase reporter assays were used to quantify KD and IC_50_ values to assess functional neutralization. This validated the consistency between the designed and actual results.

### 2.6 Circular Dichroism (CD) Measurement

CD measurements were conducted using a JASCO J1500 spectrometer to evaluate the secondary structure stability of the binder proteins. The measurements were performed across a temperature range of 25°C–95°C using a 1 mm path‐length cuvette. The wavelength range was set between 200 and 260 nm, covering the characteristic peptide backbone absorptions in the far‐ultraviolet (UV) region. Proteins were prepared at a concentration of 0.4 mg/mL in PBS buffer (pH 7.4). Samples were equilibrated for 5 min at each temperature before measurements to ensure thermal equilibrium. CD spectra were recorded in 3°C increments from 25°C to 95°C. Each spectrum was averaged over 3 scans to improve signal‐to‐noise ratio. Thermal denaturation curves were obtained by measuring the CD signal at 222 nm (typical for α‐helix content) as the temperature increased. The melting temperature (Tm), representing the temperature at which the protein undergoes significant unfolding, was determined from the thermal transition. The measurements were carried out on a JASCO J1500 spectrometer, equipped with a temperature‐controlled Peltier system for precise temperature regulation.

### 2.7 Cell Lines and GDF15‐Responsive Signaling Models

For in vitro GDF15–GFRAL signaling assays, we used HEK293 cells stably expressing human GFRAL and its co‐receptor RET, along with SRE‐Luc2 luciferase reporter gene, a widely used and well‐established system in which GDF15 robustly activates downstream ERK signaling and transcriptional responses [31]. This system provides a sensitive and reproducible readout of GDF15 pathway activity and is commonly used for functional GDF15 neutralization assays. Murine tumor cell lines used for in vivo studies included MC38 (colon adenocarcinoma), LLC1 (Lewis lung carcinoma), and A20 (B cell lymphoma). All cell lines were maintained in DMEM (MC38, LLC1) or RPMI‐1640 (A20) supplemented with 10% fetal bovine serum and penicillin–streptomycin and were confirmed to be mycoplasma‐free prior to experiments.

### 2.8 GDF15 Stimulation Conditions

For biochemical and transcriptional assays, recombinant human GDF15 was used at a final concentration of 50 ng/mL, a dose commonly reported to robustly activate GDF15–GFRAL signaling. Western blot (pERK/ERK): cells were serum‐starved for 12–16 h and stimulated with GDF15 for 15 min. qRT–PCR (immediate early genes): cells were stimulated with GDF15 for 60 min prior to RNA extraction. Luciferase reporter assays: cells were treated with GDF15 for 6 h before measurement of luciferase activity. Where indicated, minibinder or control reagents were pre‐incubated with GDF15 for 30 min at room temperature before addition to cells.

### 2.9 Western Blotting for Downstream Phosphorylation (pERK/ERK)

Cells were serum‐starved for 12‐16 h and stimulated with recombinant GDF15 (± minibinder or anti‐GDF15 antibody) for the indicated time. Cells were lysed in ice‐cold RIPA buffer supplemented with protease/phosphatase inhibitors. Lysates were clarified by centrifugation, and protein concentration was determined by BCA assay. Equal amounts of protein were resolved by SDS–PAGE, transferred to PVDF membranes, blocked in 5% BSA in TBST, and incubated with primary antibodies against phospho‐ERK1/2 (Thr202/Tyr204; CST #4370), total ERK1/2 (CST #9102), and α‐tubulin (CST #3873) overnight at 4°C. Membranes were incubated with HRP‐conjugated secondary antibodies and developed using ECL.

### 2.10 qRT–PCR for Immediate Early Genes (IEGs)

Cells were stimulated with GDF15 (± minibinder or control) for 1 h. Total RNA was extracted using a silica‐column kit or TRIzol‐based method, treated with DNase, and reverse‐transcribed to cDNA using a standard reverse transcription kit. Quantitative PCR was performed using SYBR Green chemistry on a real‐time PCR system. Target genes were normalized to housekeeping gene b‐actin. Relative expression was calculated using the 2^‐ΔΔCt^ method and presented as fold‐change vs. vehicle control.

### 2.11 Luciferase Reporter Assay for GDF15–GFRAL Pathway Activity

HEK293 cells stably expressing human GFRAL and its co‐receptor RET, along with SRE‐Luc2 luciferase reporter gene were used. HEK293 cells stably expressing human GFRAL and its co‐receptor RET, together with an integrated SRE‐Luc2 luciferase reporter gene, were used to assess GDF15–GFRAL pathway activity. For luciferase assays, cells were seeded into 96‐well plates at a density optimized to ensure logarithmic growth at the time of stimulation. Following overnight attachment, cells were serum‐starved for 4 h and then stimulated with recombinant human GDF15 (50 ng/mL) in the presence or absence of GDF15 minibinders or control reagents. Where indicated, GDF15 was pre‐incubated with minibinders for 30 min at room temperature prior to addition to cells. After 6 h of stimulation, luciferase activity was measured using a luciferase detection reagent according to the manufacturer's instructions. Luminescence was recorded on a microplate reader, and raw values were normalized to the corresponding vehicle‐treated controls. Data are presented as relative luciferase activity, reflecting SRE‐driven transcription downstream of GDF15–GFRAL signaling.

### 2.12 ELISA‐Based Competition Assay

An ELISA‐format competition assay was used to quantify inhibition of GDF15–GFRAL interaction. Briefly, one binding partner (GFRAL ectodomain) was immobilized on high‐binding plates, followed by incubation with GDF15 in the presence of serial dilutions of minibinder. Bound GDF15 was detected using an appropriate detection antibody, and inhibition curves were fit using a four‐parameter logistic model to derive IC_50_ values (log scale). For charge‐complementary rescue experiments, IC_50_ values were determined for wild‐type and mutant combinations of GDF15 and minibinders, where the charge‐complementary double mutant exhibited partial recovery of neutralization potency relative to single mutants.

### 2.13 Mice

All animal procedures were approved by the Institutional Animal Care and Use Committee of Chinese PLA General Hospital with approval number 2024‐X20‐13. Use female or male mice consistently (8 weeks), strain‐matched to tumor lines (MC38/LLC1 C57BL/6; A20 BALB/c). All mice used in this study were purchased from Charles River Laboratories and maintained under specific pathogen–free (SPF) conditions.

### 2.14 Tumor Inoculation, Monitoring and Treatments

Cells were harvested in log‐phase growth, washed, and resuspended in sterile PBS. Subcutaneous tumors were established by injection into the flank in a volume of 100 µL. MC38 2 × 10^6^ cells per mouse, LLC1 1 × 10^6^ cells per mouse. A20: 1 × 10^6^ cells. Tumor size was measured by digital calipers 2–3 times per week. Tumor volume was calculated as (length × width^2^)/2. Body weight was measured in parallel on the same schedule and plotted as absolute weight or percent change from baseline (day of randomization). Treatment was initiated when tumors reached approximately 50–150 mm^3^ in volume (typically 5–7 days after implantation). GDF15 minibinders were administered by intraperitoneal injection at a dose of 10 mg/kg every two days. Anti–PD‐1 antibody (InVivoMAb anti‐mouse PD‐1, clone RMP1‐14) was administered intraperitoneally at 5 mg/kg twice weekly.

### 2.15 Cachexia Intervention and Endpoints

Mice were randomized when tumors became palpable or reached a predefined volume (50–150 mm^3^). Minibinder (and relevant controls) was administered every 2 days. Cachexia endpoints included progressive body weight loss, clinical score, and survival. Food intake was measured at day 15 after tumor inoculation. Inguinal white adipose tissue (iWAT), gastrocnemius muscles were dissected and weighed immediately after euthanasia at day 15. Humane endpoints were pre‐defined (tumor > 2000 mm^3^, >25% body weight loss, severe morbidity). Survival was analyzed using Kaplan–Meier curves.

### 2.16 Tumor Processing for Flow Cytometry

Tumors were excised at the indicated time points and immediately placed in ice‐cold RPMI‐1640 medium. Tumor tissues were finely minced using sterile scissors and subjected to enzymatic digestion in RPMI‐1640 containing collagenase type IV (1 mg/mL) and DNase I (100 U/mL) at 37°C for 30 min with gentle agitation. Following digestion, samples were mechanically dissociated by gentle pipetting and filtered through a 70‐µm cell strainer to obtain single‐cell suspensions. Cells were washed with cold staining buffer (PBS supplemented with 2% fetal bovine serum) and red blood cells were lysed using ammonium–chloride–potassium (ACK) lysis buffer when necessary. Cell suspensions were counted, and viability was assessed prior to staining. For surface staining, cells were incubated with Fc receptor–blocking antibody (anti‐CD16/32) for 10 min at 4°C, followed by staining with fluorophore‐conjugated antibodies against surface markers, including CD8α, for 30 min at 4°C in the dark. All flow cytometry antibodies were obtained from BioLegend. For intracellular cytokine staining, cells were restimulated ex vivo with phorbol 12‐myristate 13‐acetate (PMA, 50 ng/mL) and ionomycin (500 ng/mL) in the presence of a protein transport inhibitor, brefeldin A, for 4 h at 37°C. Cells were then fixed and permeabilized using a commercial fixation/permeabilization kit according to the manufacturer's instructions, followed by intracellular staining with BioLegend antibodies against IFN‐γ and granzyme B. Data were acquired on a flow cytometer and analyzed using FlowJo software. Gating was performed sequentially on singlets, live cells, lymphocytes, CD8^+^ T cells, and cytokine‐positive populations.

### 2.17 Immunofluorescence

Tumors were harvested, fixed with 4% paraformaldehyde followed by cryoprotection, sectioned, and subjected to antigen retrieval if required. Sections were blocked (5% normal serum + 0.1% Triton X‐100), incubated with primary antibody against CD8α (CST #85336) overnight at 4°C, washed, and incubated with fluorophore‐conjugated secondary antibody. Nuclei were counterstained with DAPI. Images were acquired with a fluorescence or confocal microscope under identical exposure settings across groups. CD8 infiltration was quantified as CD8^+^ area fraction and/or CD8^+^ cell counts per field in a blinded fashion.

### 2.18 Statistical Analysis

Data are presented as mean ± s.d. unless otherwise indicated. Comparisons between two groups were performed using two‐tailed Student's *t*‐test; multiple group comparisons used one‐way or two‐way ANOVA with appropriate multiple comparison correction. Survival was analyzed by log‐rank (Mantel–Cox) test. IC_50_ values were obtained by nonlinear regression (four‐parameter logistic model). In all figures, ns indicates not significant. Statistical significance was defined as follows: ^*^ *p* < 0.05, ^**^ *p* < 0.01, ^***^ *p* < 0.001, and ^****^ *p* < 0.0001.

## 3 Results

### 3.1 Structure‐Guided De Novo Design of Minibinders Targeting the GDF15–GFRAL Interface

**FIGURE 1:**
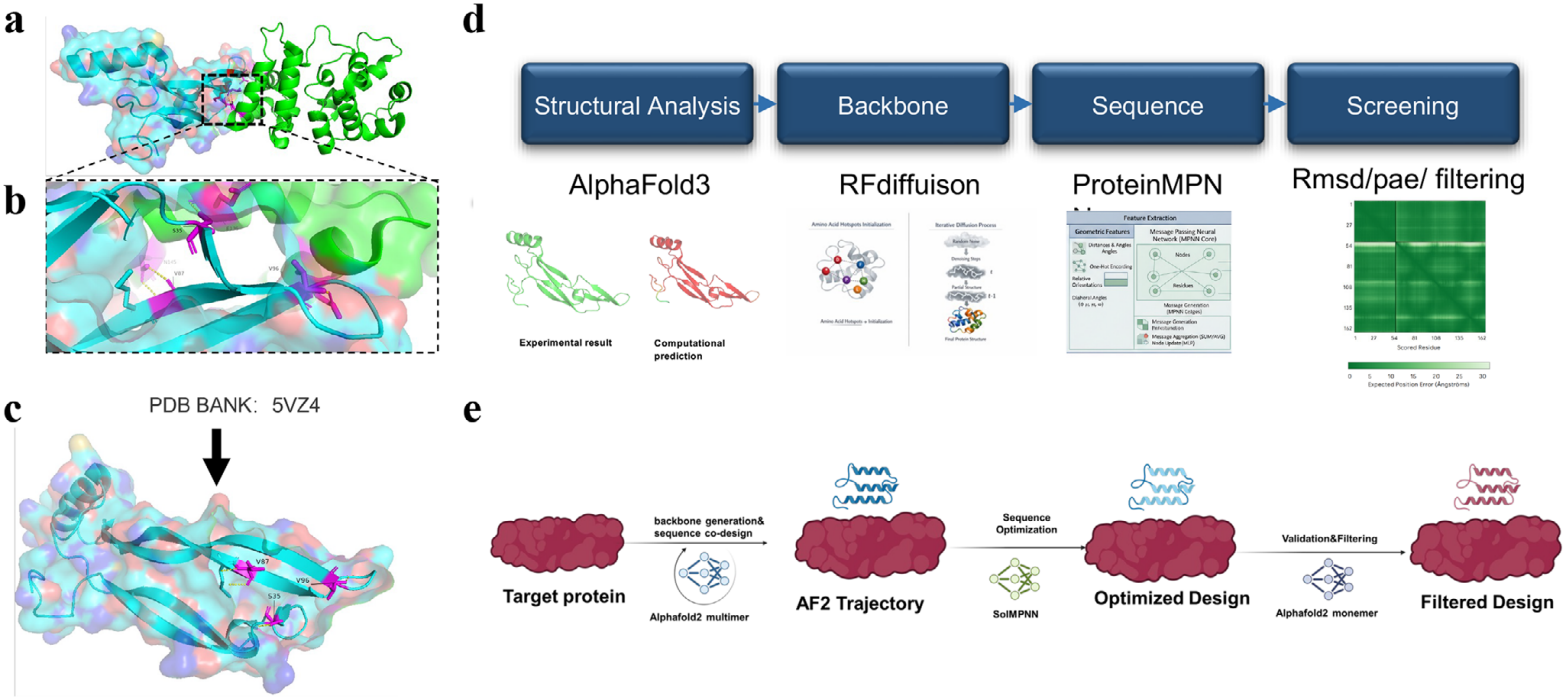
Structure‐guided de novo design of minibinders targeting the GDF15–GFRAL interface. (a) The overall structure of the GDF15/GFRAL complex, highlighting the spatial interaction between GDF15 (green) and GFRAL (cyan). (b) A close‐up view of the interface between GDF15 and GFRAL, showing critical residues involved in binding. These residues are labeled and highlighted in purple. (c) A rotated view of the complex, further illustrating the interaction between the two proteins. The purple‐labeled residues represent the hotspot residues for binder design, with structural details from the PDB bank (5VZ4) annotated. (d) GDF15 antagonistic binder design pipeline based on RFdiffusion. This includes structural analysis with AlphaFold3, followed by backbone and sequence optimization with RFdiffusion and ProteinMPNN, and subsequent screening and validation based on RMSD and PAE. (e) Binder design process based on Bindercraft. The process begins with the target protein, proceeds through co‐design with AlphaFold2 multimer, sequence optimization, and ends with validation to obtain the final filtered binder design.

To rationally disrupt GDF15–GFRAL signaling, we first performed a structure‐guided analysis of the GDF15–GFRAL interaction interface using available high‐resolution structural information. Inspection of the GDF15–GFRAL complex revealed a well‐defined receptor‐binding surface on GDF15 characterized by a concave topology and clustered interaction hotspots, suggesting suitability for targeted protein binder design (Figure 1a,b). Structural mapping of this interface onto the experimentally determined GDF15 structure (PDB ID: 5VZ4) enabled precise delineation of residues contributing to receptor engagement, which served as the foundation for subsequent computational design efforts (Figure 1c).

We next established an integrated computational pipeline to generate de novo minibinders that selectively target the GDF15–GFRAL interface (Figure 1d). Structural analysis and complex modeling were performed using AlphaFold‐based prediction, providing a consistent reference for interface geometry. Backbone generation was carried out with RFdiffusion, which enabled the creation of diverse binding scaffolds geometrically complementary to the target surface. Candidate backbones were subsequently subjected to sequence optimization using ProteinMPNN, yielding sequences predicted to stabilize the designed binding conformations. To ensure structural fidelity and binding plausibility, designs were filtered based on multiple criteria, including predicted alignment error (PAE) and structural deviation metrics.

In parallel, we implemented an iterative design workflow that couples backbone generation, sequence co‐design, and structure prediction to progressively refine minibinder candidates (Figure 1e). Each design trajectory was evaluated for monomeric stability and interface compatibility using AlphaFold‐based structure prediction, allowing early elimination of unstable or poorly folded candidates. This multi‐stage filtering strategy resulted in a focused set of high‐confidence minibinder designs predicted to engage the GDF15 receptor‐binding surface with high structural complementarity, forming the basis for downstream experimental validation.

### 3.2 Computational Selection and Structural Characterization of High‐Confidence GDF15 Minibinders

**FIGURE 2:**
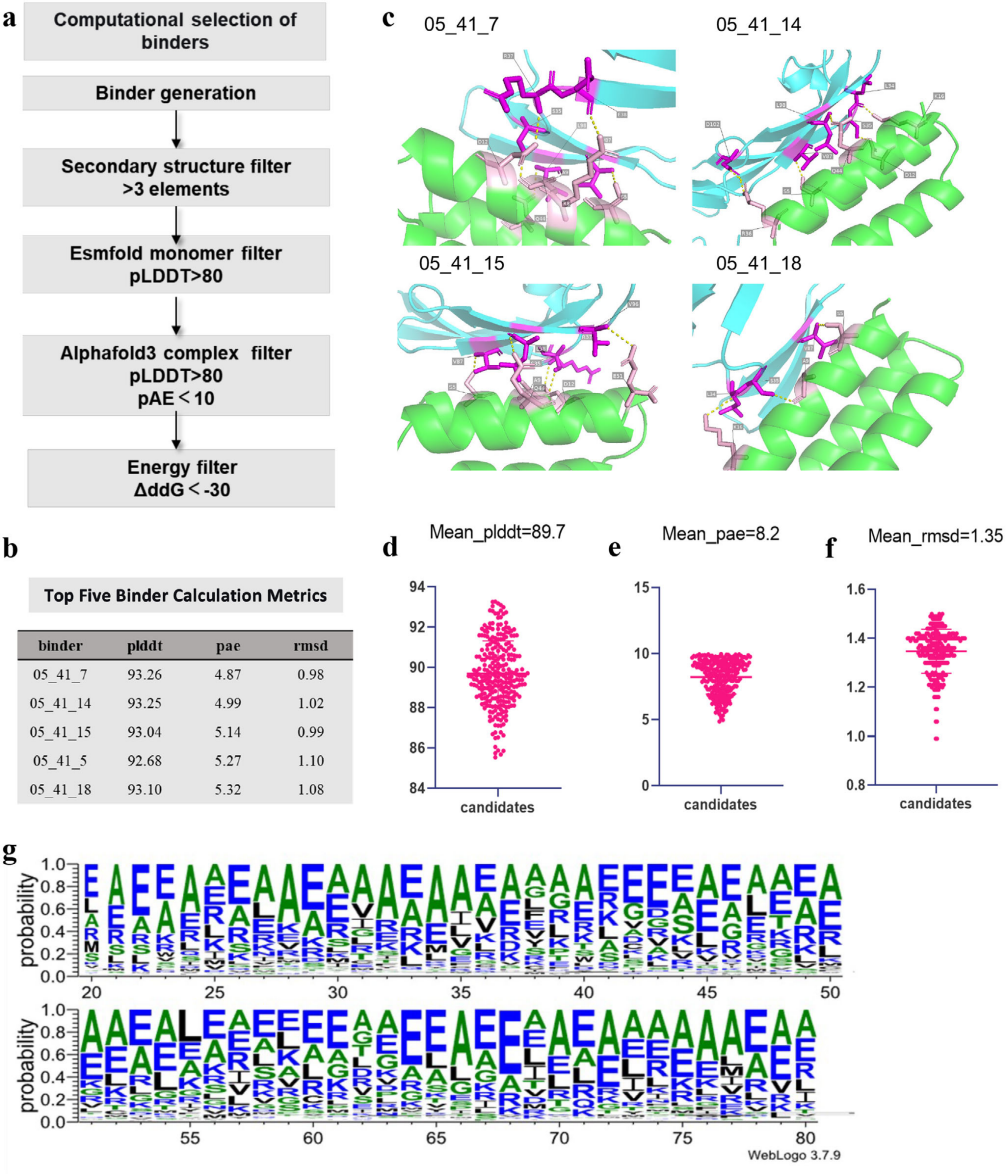
Optimization of the binders based on pAE interaction scores. (a) The computational binder selection pipeline. Binders are initially generated and filtered by secondary structure, requiring more than three structural elements. This is followed by an Esmdfold monomer filter (pLDDT > 80), an AlphaFold 3 complex filter (pLDDT > 80, pAE < 10), and an energy filter (ΔΔG < −30) to identify high‐quality candidates. (b) Top five binder calculation metrics. The pLDDT, pAE, and RMSD values of the top five binders are presented, highlighting the computational performance of each binder. (c) Structural representations of the top binders. The left and right panels show detailed views of the interface between the binder (green) and GDF15 (cyan), with key interaction residues highlighted in purple. Residues in cyan correspond to predicted key residues on GDF15, while those in pink correspond to critical residues on the binder. (d‐f) Statistical analysis of binder performance. Mean pLDDT, pAE, and RMSD values are displayed for the candidates, representing the overall computational quality of the binder designs. (g) Sequence analysis of the binders. The WebLogo representation shows the amino acid distribution at each position across all candidate binders, with the probability of each amino acid at every position, facilitating sequence feature analysis of the optimized binders.

Following structure‐guided targeting of the GDF15–GFRAL interface, we generated a large pool of de novo minibinder candidates using our integrated computational design pipeline. To systematically enrich for structurally robust and interface‐compatible binders, candidates were subjected to a multi‐stage filtering process incorporating secondary structure complexity, monomeric fold confidence, complex prediction quality, and energetic favorability (Figure 2a). Specifically, designs containing more than three secondary structure elements were retained to favor compact and stable folds, followed by EsmFold‐based monomer filtering to exclude poorly folded candidates (pLDDT > 80). Surviving designs were further evaluated in complex with GDF15 using AlphaFold3, and filtered based on both local confidence (complex pLDDT > 80) and interface geometry (predicted alignment error, PAE < 10). Finally, designs were ranked by predicted binding energetics, retaining candidates with strongly favorable interaction energies (ΔΔG < −30).

This hierarchical filtering strategy yielded a focused subset of high‐confidence minibinders exhibiting consistent structural quality across multiple metrics. The top‐ranking designs displayed high predicted fold confidence and interface accuracy, with representative candidates showing mean pLDDT values of approximately 90, low predicted alignment errors, and minimal deviation between designed and predicted structures (Figure 2b,d–f). Notably, the convergence of independent quality metrics suggested robust sequence–structure compatibility rather than overfitting to any single evaluation criterion.

Structural inspection of the top minibinders revealed well‐defined binding modes at the GDF15 receptor‐interaction surface. Representative AlphaFold3‐predicted complexes demonstrated that distinct minibinder scaffolds docked into the targeted interface with high shape complementarity and consistent engagement of key hotspot residues (Figure 2c). Despite diversity in backbone topology, these designs converged on a shared binding surface, indicating that the computational pipeline successfully captured the dominant geometric and chemical features required for GDF15 recognition.

Analysis of sequence variability across high‐confidence designs further revealed conserved positional preferences within the minibinder cores and interface‐contacting regions (Figure 2g). Sequence logos derived from the filtered design set showed strong enrichment of specific residue classes at structurally constrained positions, consistent with stabilization of the designed fold and optimization of interface interactions. In contrast, solvent‐exposed regions exhibited greater sequence diversity, suggesting tolerance for variation without compromising structural integrity.

Together, these results demonstrate that the integrated design and filtering pipeline efficiently enriches for de novo minibinders with high predicted structural fidelity, favorable binding energetics, and convergent recognition of the GDF15 receptor‐binding surface. This computationally selected minibinder set formed the basis for subsequent experimental validation and functional characterization.

### 3.3 Experimental Validation Identifies High‐Affinity GDF15 Minibinders

**FIGURE 3:**
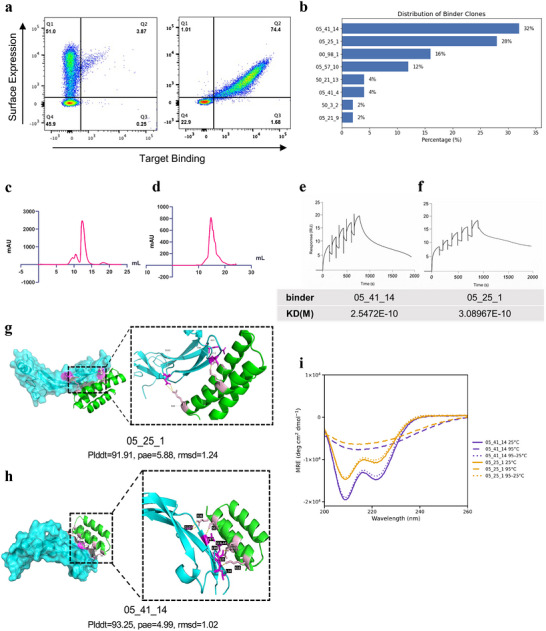
Biochemical Characterization of GDF15 Antagonistic Binders. (a) Yeast surface display analysis showing surface expression vs. GDF15 target binding for representative minibinder clones. Quadrant gating identifies populations exhibiting both high expression and strong target binding. (b) Distribution of enriched binder clones following yeast display selection, determined by deep sequencing. Percentages indicate the relative abundance of individual designs within the selected population. (c,d) Size‐exclusion chromatography profiles of purified minibinders 05_25_1 (c) and 05_41_14 (d), showing monomeric elution behavior consistent with stable folding. (e,f) Surface plasmon resonance sensorgrams for minibinders 05_41_14 (e) and 05_25_1 (f) binding to GDF15. Minibinders were injected at 3.125, 6.25, 12.5, 25, and 50 nm, and kinetic parameters were obtained by global fitting to a 1:1 Langmuir binding model. KD values are shown as representative values from independent measurements. Fitted curves indicate sub‐nanomolar affinities. (g,h) AlphaFold3‐predicted structures of minibinder–GDF15 complexes for 05_25_1 (g) and 05_41_14 (h). Insets highlight the binding interfaces. Predicted confidence metrics (pLDDT, PAE, RMSD) are indicated.

To experimentally validate the computationally designed GDF15 minibinders, we expressed the filtered candidate library on the yeast cell surface and assessed both target binding and surface expression by flow cytometry. Yeast display analysis revealed a clear population of clones exhibiting robust GDF15 binding while maintaining high surface expression, enabling efficient discrimination of functional binders from poorly expressed or non‐binding variants (Figure 3a).

Deep sequencing of the enriched yeast populations revealed a highly non‐uniform distribution of binder clones, with a small number of designs dominating the selected pool (Figure 3b). Notably, several top‐ranking designs, including clones 05_41_14 and 05_25_1, accounted for a substantial fraction of the total enriched population, indicating strong selection pressure favoring specific minibinder architectures. To further characterize the biochemical properties of these lead candidates, representative binders were recombinantly expressed and purified to homogeneity, as confirmed by size‐exclusion chromatography (Figure 3c,d). Both binders eluted as single, symmetric peaks consistent with monomeric species, suggesting favorable folding and solution stability.

Binding kinetics were next quantified using surface plasmon resonance (SPR). Both lead minibinders bound GDF15 with sub‐nanomolar affinities, displaying rapid association and slow dissociation kinetics (Figure 3e,f). The equilibrium dissociation constants were determined to be 2.5 × 10‐10 m for clone 05_41_14 and 3.1 × 10‐10 m for clone 05_25_1, confirming that de novo designed minibinders can achieve high‐affinity recognition of GDF15. Structural inspection of AlphaFold3‐predicted minibinder–GDF15 complexes revealed close agreement between designed and predicted binding modes (Figure 3g,h). Both minibinders engaged the targeted receptor‐binding surface on GDF15 with high shape complementarity and consistent positioning of interface residues. Quantitative analysis showed low root‐mean‐square deviations (RMSD ≈ 1 Å), low predicted alignment errors (PAE < 6), and high pLDDT scores (> 90), supporting the structural fidelity of the designs.

Finally, circular dichroism spectroscopy demonstrated that the minibinders adopted stable secondary structures across a range of temperatures (Figure 3i). Thermal unfolding profiles indicated minimal loss of structural integrity up to 95°C, highlighting the exceptional thermostability of the designed binders.

Together, these results establish that the computational pipeline yields de novo minibinders that are not only structurally accurate but also biochemically stable and capable of binding GDF15 with picomolar affinity, providing a robust foundation for downstream functional and in vivo studies.

### 3.4 De Novo Minibinders Competitively Block the GDF15–GFRAL Interaction Through Defined Interface Contacts

**FIGURE 4:**
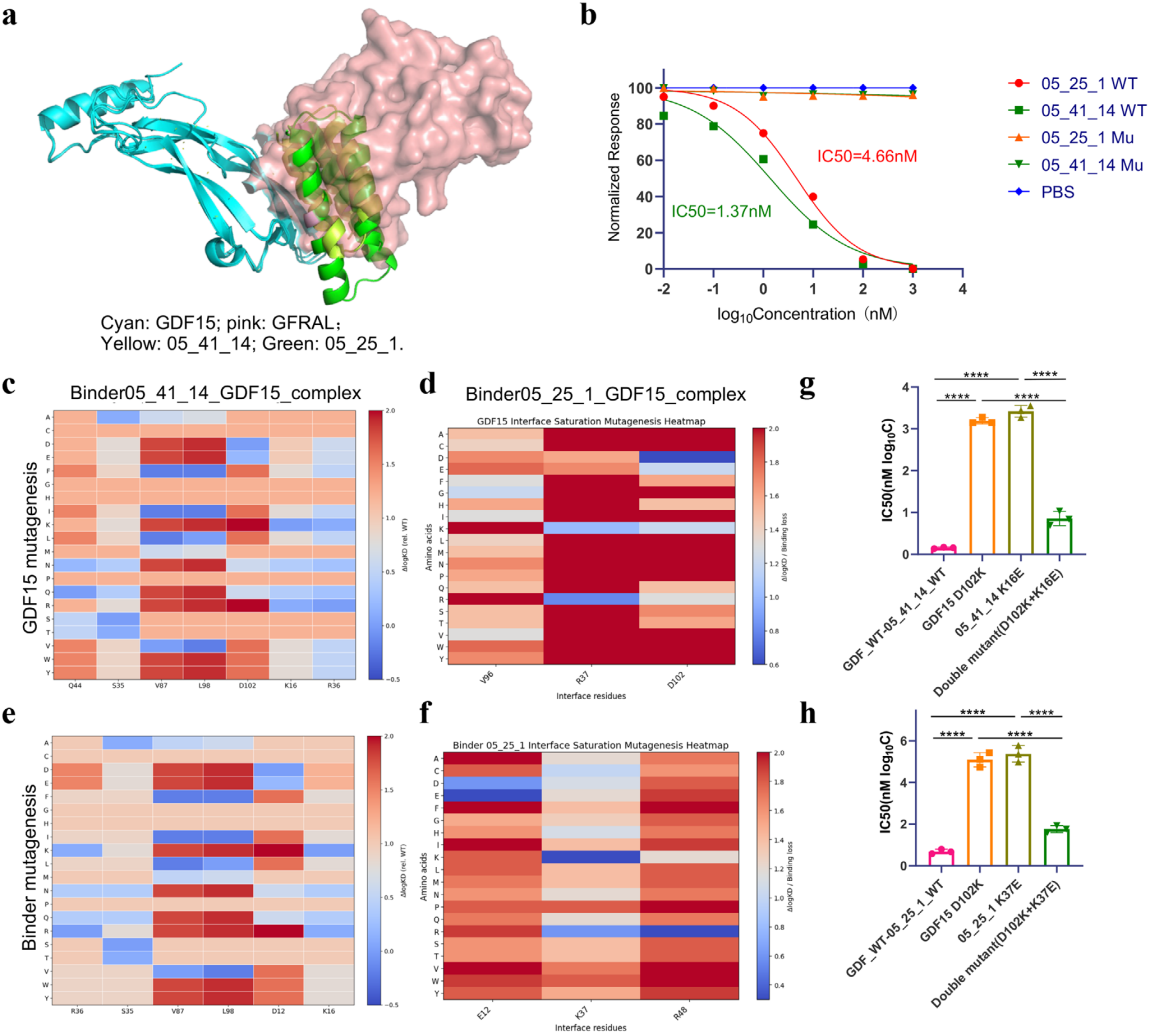
De Novo Minibinders Competitively Block the GDF15–GFRAL Interaction. (a) Structural superposition of minibinder–GDF15 complexes with the GDF15–GFRAL complex. GDF15 is shown in cyan, GFRAL in pink, minibinder 05_41_14 in yellow, and minibinder 05_25_1 in green, illustrating direct overlap between minibinder binding sites and the GFRAL interaction interface. (b) ELISA‐based competition assay was performed at a fixed concentration of GDF15 (5 nm). Dose–response inhibition curves showing suppression of GDF15 activity by minibinders 05_41_14 and 05_25_1. Wild‐type minibinders exhibit low‐nanomolar IC_50_ values, whereas interface‐mutant variants show reduced inhibitory potency. Data are shown as mean ± s.d. (c,d) Heat map summarizing the effects of amino‐acid substitutions at predicted GDF15 interface residues on binding to Binder 05_41_14 (c) and 05_25_1(d). Changes in binding affinity are expressed as ΔlogKD relative to wild type, as determined by SPR. Red indicates reduced binding affinity, whereas blue indicates minimal loss of binding. (e,f) Heat map showing the impact of site‐saturation mutagenesis at Binder 05_41_14(e) and 05_25_1(f) interface residues on GDF15 binding. Mutations at central interface positions result in pronounced losses of binding affinity, consistent with a localized and well‐defined interaction interface. (g,h) Charge‐complementary rescue mutations restore functional neutralization. IC_50_ values (log scale) derived from the ELISA‐based competition assay for wild‐type and mutant combinations of GDF15 and the minibinders. Charge‐disrupting single mutations on either partner reduce neutralization potency, whereas the charge‐complementary double‐mutant combination shows partial recovery of neutralization activity, consistent with a specific electrostatic interaction at the designed interface.

Given the high affinity and structural fidelity of the lead minibinders, we next investigated whether these molecules directly interfere with the GDF15–GFRAL interaction. Structural superposition of AlphaFold3‐predicted complexes revealed that both minibinders bind to the receptor‐facing surface of GDF15, occupying the GFRAL interaction interface (Figure 4a). The predicted binding modes showed extensive spatial overlap between minibinders and GFRAL, indicating that minibinder binding is incompatible with receptor engagement and suggesting a competitive mechanism of inhibition.

To quantitatively assess functional blockade, we measured the ability of minibinders to inhibit GDF15‐induced signaling using a dose–response assay. Both 05_41_14 and 05_25_1 efficiently suppressed GDF15 activity in a concentration‐dependent manner, with half‐maximal inhibitory concentrations (IC_50_) in the low nanomolar range (Figure 4b). In contrast, mutations introduced at predicted interface residues of either GDF15 or the minibinders substantially reduced inhibitory potency, confirming the importance of the designed interaction surfaces.

To systematically map the molecular determinants of binding, we performed saturation mutagenesis of interface residues on GDF15 and assessed their effects on minibinder binding. Heatmap analysis revealed distinct clusters of residues whose mutation selectively impaired binding of either 05_41_14 or 05_25_1, consistent with partially overlapping but non‐identical binding footprints on GDF15 (Figure 4c,d). Notably, mutations at key hotspot residues resulted in pronounced loss of inhibition, underscoring their central role in minibinder engagement.

Reciprocal saturation mutagenesis of minibinder interface residues further validated the designed binding modes (Figure 4e,f). Mutations disrupting core interface contacts led to marked reductions in inhibitory activity, whereas substitutions at peripheral positions were better tolerated. These data indicate that the minibinders rely on a defined set of interface residues to achieve high‐affinity binding and functional blockade.

To further test whether minibinder neutralization depends on the specific electrostatic contacts predicted at the interface, we designed charge‐disrupting mutations on GDF15 and the binders and quantified functional neutralization using an ELISA‐based competition assay. Single charge‐reversal mutations on either GDF15 or the minibinders markedly reduced neutralization potency, shifting IC_50_ values to higher concentrations (Figure 4g,h). Strikingly, introducing charge‐complementary rescue mutations—pairing the GDF15 mutation with the corresponding compensatory mutation on the minibinder—partially restored neutralization potency, as evidenced by a leftward shift of the IC_50_ relative to the non‐complementary single‐mutant combinations (Figure 4g,h). This “charge‐swap” rescue provides strong functional evidence that the minibinders neutralize GDF15 through the intended, structure‐defined interface rather than nonspecific binding, and supports a competitive mechanism of inhibition at the GDF15–GFRAL interaction surface.

### 3.5 Minibinder‐Mediated Neutralization of GDF15 Suppresses Downstream Signaling and Reverses Cancer‐Associated Cachexia In Vivo

**FIGURE 5:**
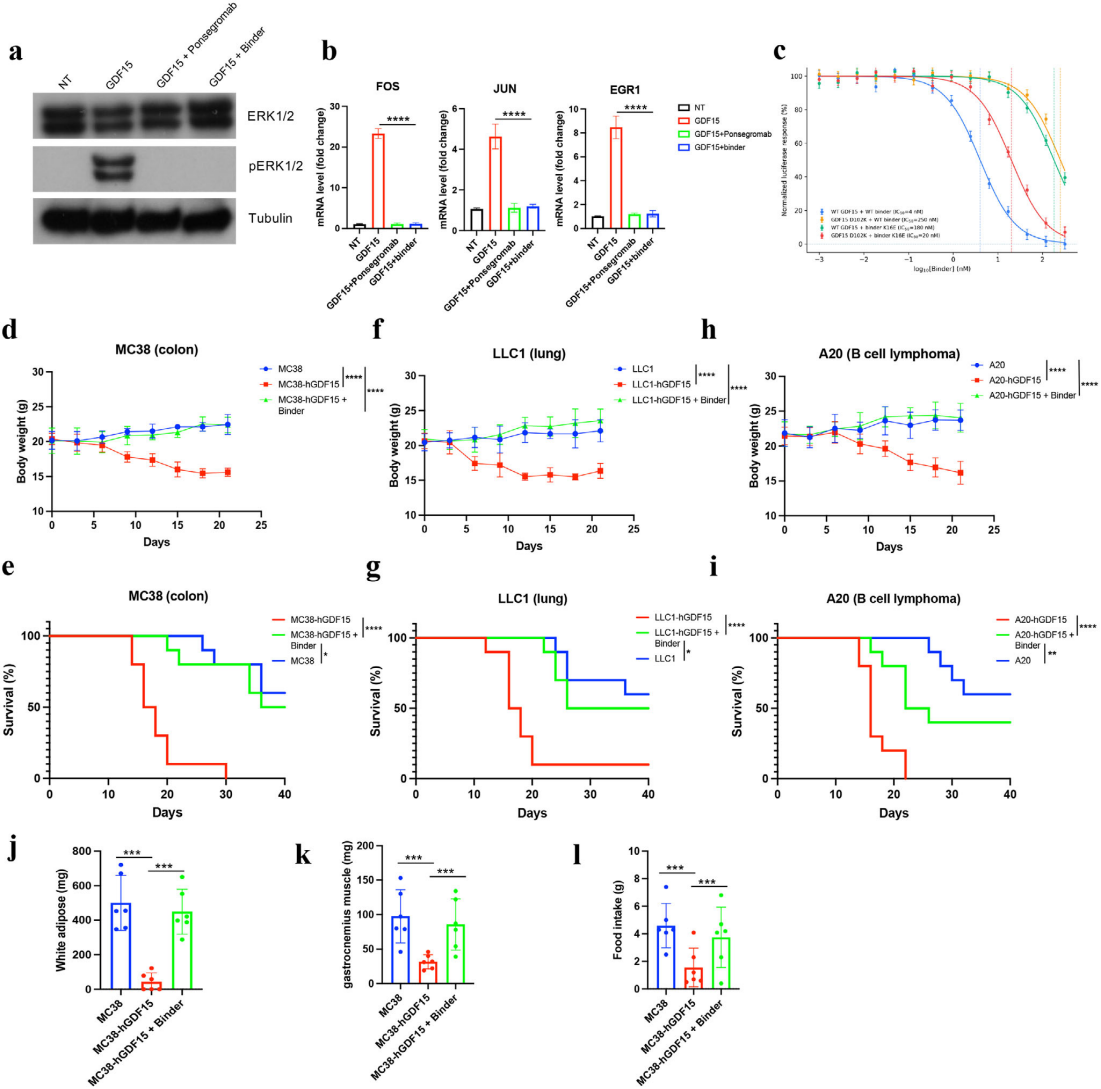
De novo GDF15 minibinders suppress downstream signaling and reverse cancer‐associated cachexia in vivo. (a) Immunoblot analysis of ERK1/2 and phosphorylated ERK1/2 (pERK1/2) following treatment with GDF15 alone or in combination with ponsegromab or the GDF15 minibinder. Tubulin serves as a loading control. (b) Quantitative RT–PCR analysis of immediate early genes (*FOS*, *JUN*, *EGR1*) following GDF15 stimulation with or without ponsegromab or the GDF15 minibinder. Data are shown as mean ± s.e.m. (c) Functional validation of the predicted electrostatic interaction interface by SRE‐luciferase assay. HEK293 cells stably expressing human GFRAL, RET, and an SRE‐Luc2 reporter were stimulated with WT or mutant GDF15 in the presence of increasing concentrations of WT or mutant minibinders. (d,f,h) Body weight trajectories of mice bearing MC38 colon (d), LLC1 lung (f), or A20 B cell lymphoma (h) tumors expressing human GDF15, with or without minibinder treatment. (e,g,i) Kaplan–Meier survival analysis of mice bearing MC38 (e), LLC1 (g), or A20 (i) GDF15‐expressing tumors. Minibinder treatment significantly prolongs survival compared with untreated GDF15‐expressing tumors (*n* = 10). (j) Quantification of white adipose tissue mass in MC38, MC38‐hGDF15, and MC38‐hGDF15 + Binder tumor‐bearing mice at endpoint. (k) Quantification of gastrocnemius muscle mass at endpoint. (l) Food intake measurements in indicated groups. MC38‐hGDF15 tumor‐bearing mice exhibited severe adipose tissue depletion, skeletal muscle wasting, and reduced food intake, all of which were significantly improved following minibinder treatment. Data are presented as mean ± SD. Statistical significance was determined using one‐way ANOVA with Tukey's multiple comparisons test. ^***^ *p* < 0.001.

To determine whether the de novo GDF15 minibinders functionally inhibit downstream signaling, we first examined activation of canonical GDF15–GFRAL pathways in vitro. Treatment with recombinant GDF15 robustly induced ERK1/2 phosphorylation, whereas co‐treatment with either the clinical anti‐GDF15 antibody ponsegromab or the designed minibinder markedly reduced pERK1/2 levels without affecting total ERK expression (Figure 5a). These data indicate that the minibinder efficiently suppresses proximal GDF15 signaling. Consistent with inhibition of ERK pathway activation, GDF15 stimulation strongly upregulated immediate early response genes, including *FOS*, *JUN*, and *EGR1*. Quantitative RT–PCR analysis revealed that both ponsegromab and the minibinder significantly attenuated GDF15‐induced transcriptional responses, restoring expression levels close to baseline (Figure 5b). These findings confirm effective blockade of GDF15 signaling at both signaling and transcriptional levels.

To functionally evaluate inhibition of GDF15 signaling through the interface, we employed an SRE‐luciferase reporter system in HEK293 cells stably expressing human GFRAL and its co‐receptor RET. Upon GDF15 stimulation, activation of the GFRAL–RET complex induces downstream MAPK/ERK signaling, which in turn activates the serum response element (SRE) reporter. Thus, SRE‐luciferase activity serves as a functional readout of GDF15–GFRAL–RET pathway activation. Consistent with the structural model, the WT minibinder potently inhibited signaling induced by WT GDF15 (IC_50_ = 4 nm). In contrast, disruption of the predicted electrostatic interaction through either the GDF15 D102K mutation or the minibinder K16E mutation markedly reduced inhibitory potency (Figure 5c). Importantly, simultaneous charge‐swapping mutations partially restored minibinder activity, supporting the existence of a specific electrostatic interaction between GDF15 D102 and minibinder residue K16. These results provide functional evidence validating the predicted binding interface and demonstrate that minibinder‐mediated antagonism depends on defined residue‐level molecular interactions.

We next assessed the physiological consequences of GDF15 neutralization in vivo using multiple tumor‐associated cachexia models. In mice bearing MC38 colon tumors engineered to express human GDF15, progressive body weight loss was observed despite ongoing tumor growth, consistent with cachexia. Administration of the GDF15 minibinder significantly mitigated weight loss, restoring body weight trajectories toward those observed in control MC38 tumors lacking GDF15 overexpression (Figure 5d). Similar protective effects were observed in LLC1 lung cancer and A20 B cell lymphoma models, demonstrating that minibinder‐mediated rescue of cachexia is not restricted to a single tumor type (Figure 5f,h). Importantly, reversal of cachexia translated into improved survival outcomes. Kaplan–Meier analysis revealed that mice bearing GDF15‐expressing tumors exhibited markedly reduced survival compared with controls, whereas treatment with the GDF15 minibinder significantly prolonged survival across all three tumor models (Figure 5e,g,i). Notably, a substantial fraction of minibinder‐treated mice remained alive at the experimental endpoint, highlighting the durable physiological benefit of GDF15 neutralization.

To further characterize the anti‐cachectic effects of GDF15 minibinder treatment, we next evaluated adipose tissue loss, skeletal muscle wasting, and food intake in the MC38‐hGDF15 tumor model after treatments. Consistent with the pronounced body‐weight reduction observed in MC38‐hGDF15 tumor‐bearing mice, these animals exhibited severe depletion of white adipose tissue and marked gastrocnemius muscle wasting compared with control MC38 tumor‐bearing mice (Figure 5j,k). Importantly, treatment with the GDF15 minibinder significantly preserved both adipose tissue mass and skeletal muscle mass, indicating effective alleviation of cachexia‐associated tissue wasting. In addition, MC38‐hGDF15 tumor‐bearing mice displayed substantially reduced food intake, consistent with the anorexigenic effects of GDF15 signaling. Minibinder treatment significantly restored food consumption toward levels observed in control mice (Figure 5l), further supporting functional blockade of the GDF15–GFRAL axis in vivo.

Together, these results demonstrate that de novo designed GDF15 minibinders effectively suppress GDF15–GFRAL signaling, reverse tumor‐induced cachexia, and confer a survival advantage across multiple cancer models.

### 3.6 GDF15 Neutralization Restores Responsiveness to Anti–PD‐1 Therapy Through CD8^+^ T Cell–Dependent Mechanisms

**FIGURE 6:**
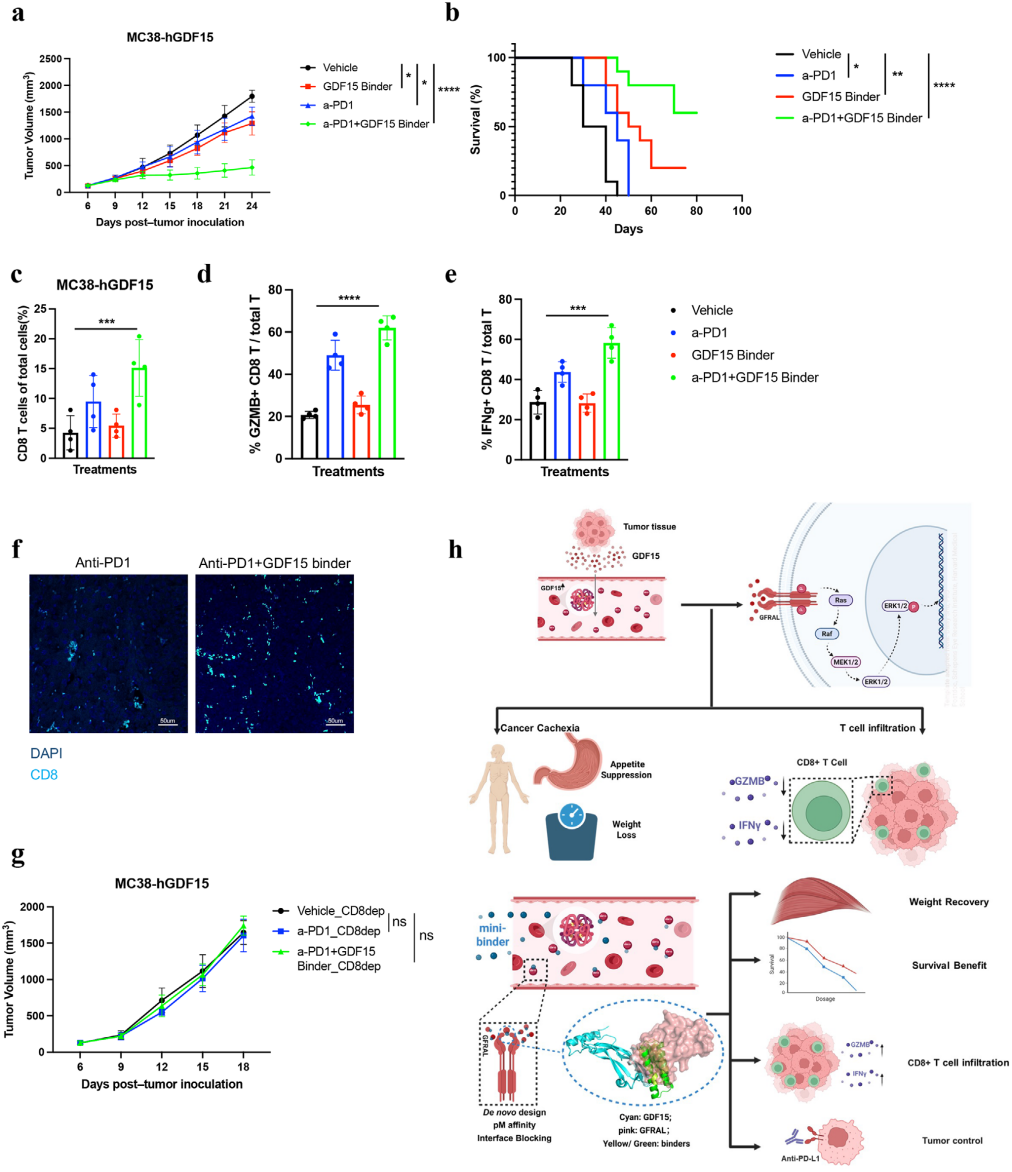
GDF15 neutralization restores sensitivity to anti–PD‐1 therapy in a CD8^+^ T cell–dependent manner. (a) Tumor growth curves of MC38‐hGDF15 tumors treated with vehicle, GDF15 minibinder, anti–PD‐1, or the combination of anti–PD‐1 (*n* = 5, 5 mg/kg, twice a week) and GDF15 minibinder (*n* = 5, 10 mg/kg, every 2 days). Data are shown as mean ± s.e.m. (b) Kaplan–Meier survival analysis of MC38‐hGDF15 tumor–bearing mice receiving the indicated treatments (*n* = 10). (c) Flow cytometric quantification of CD8^+^ T cells as a percentage of total tumor‐infiltrating cells under the indicated treatment conditions (*n* = 4). (d,e) Frequencies of IFNγ^+^ (d) and GZMB^+^ (e) CD8^+^ T cells among total intratumoral T cells, assessed by flow cytometry. (f) Representative immunofluorescence images of MC38‐hGDF15 tumor sections stained for CD8 (cyan) and DAPI (blue) following treatment with anti–PD‐1 alone or anti–PD‐1 plus GDF15 minibinder. Scale bar, 50 µm. (g) Tumor growth curves of MC38‐hGDF15 tumors under CD8^+^ T cell depletion, showing loss of therapeutic efficacy of the anti–PD‐1 and GDF15 minibinder combination. (h) Model illustrating coordinated metabolic and immune restoration by de novo designed GDF15 minibinders. Tumor‐derived GDF15 is released into the circulation and engages its brainstem receptor GFRAL to suppress appetite and promote weight loss through downstream MAPK signaling, driving cancer‐associated cachexia. In parallel, elevated GDF15 within the tumor microenvironment limits CD8^+^ T‐cell infiltration, resulting in reduced intratumoral effector activity and impaired responsiveness to immune checkpoint blockade. De novo‐designed GDF15 minibinders with picomolar affinity block a dominant functional interface on GDF15, preventing receptor engagement and neutralizing GDF15 activity. This ligand‐level neutralization alleviates cachexia‐associated physiological decline, restores CD8^+^ T‐cell infiltration and effector function, and enables effective antitumor responses to anti–PD‐1 therapy.

Given recent evidence linking GDF15 to resistance to immune checkpoint blockade [13, 14], we next asked whether neutralization of GDF15 with binder could restore sensitivity to anti–PD‐1 therapy in a GDF15‐driven tumor model. Using MC38 tumors engineered to express human GDF15, we observed that anti–PD‐1 monotherapy only modestly delayed tumor growth, consistent with a state of immunotherapy resistance (Figure 6a). In contrast, combined treatment with anti–PD‐1 and the GDF15 minibinder resulted in a pronounced suppression of tumor growth, significantly outperforming either monotherapy (Figure 6a).

This synergistic antitumor effect translated into a marked survival benefit. Kaplan–Meier analysis showed that mice receiving the combination of anti–PD‐1 and GDF15 minibinder exhibited substantially prolonged survival compared with vehicle, anti–PD‐1 alone, or minibinder alone (Figure 6b). Notably, a large fraction of mice in the combination group remained alive at the experimental endpoint, indicating durable therapeutic efficacy.

To investigate the immunological basis of this synergy, we analyzed tumor‐infiltrating lymphocytes by flow cytometry. Combination therapy significantly increased the frequency of CD8^+^ T cells within the tumor microenvironment compared with all other treatment groups (Figure 6c). Moreover, CD8^+^ T cells from tumors treated with anti–PD‐1 plus GDF15 minibinder displayed enhanced effector function, as evidenced by increased expression of interferon‐γ (IFNγ) and granzyme B (GZMB) (Figure 6d,e). Consistent with these findings, immunofluorescence staining of tumor sections revealed increased CD8^+^ T cell infiltration in tumors treated with the combination therapy relative to anti–PD1 alone (Figure 6f), supporting a role for GDF15 neutralization in promoting cytotoxic T cell access to the tumor.

To directly test whether the antitumor effect of combination therapy was dependent on CD8^+^ T cells, we performed antibody‐mediated CD8 depletion. Under CD8‐depleted conditions, the therapeutic benefit of anti–PD‐1 plus GDF15 minibinder was completely abolished, with tumor growth kinetics indistinguishable from those observed in control groups (Figure 6g). These data demonstrate that restoration of anti–PD‐1 efficacy by GDF15 neutralization is strictly dependent on CD8^+^ T cells.

To integrate the metabolic and immunological effects of GDF15 neutralization, we developed a working model summarizing how tumor‐derived GDF15 shapes systemic physiology and antitumor immunity and how de novo designed minibinders intervene in this process (Figure 6h). In GDF15‐high tumors, elevated circulating GDF15 engages its brainstem receptor GFRAL to suppress appetite and promote weight loss, driving cancer‐associated cachexia through activation of downstream MAPK signaling. In parallel, tumor‐derived GDF15 acts locally within the tumor microenvironment to limit CD8^+^ T‐cell infiltration, resulting in reduced intratumoral effector activity and impaired responsiveness to immune checkpoint blockade. Neutralization of GDF15 by de novo designed minibinders directly targeting a dominant functional interface on the ligand effectively blocks GDF15–GFRAL signaling and alleviates cachexia‐associated physiological decline. At the same time, GDF15 neutralization restores CD8^+^ T‐cell access to the tumor, leading to increased intratumoral accumulation of IFN‐γ– and granzyme B–producing cytotoxic T cells. This relief of immune exclusion enables anti–PD‐1 therapy to exert antitumor efficacy in GDF15‐expressing tumors that are otherwise resistant to immune checkpoint blockade.

Together, these results establish that GDF15‐driven cachexia is associated with impaired antitumor immunity and resistance to immune checkpoint blockade, and that de novo designed GDF15 minibinders can restore anti–PD‐1 responsiveness through CD8^+^ T cell–mediated mechanisms.

## 4 Discussion

Cancer cachexia remains a major unmet clinical challenge, contributing substantially to morbidity, mortality, and resistance to anticancer therapies [32, 33]. Although GDF15 has emerged as a central driver of cancer‐associated cachexia through its action on the brainstem receptor GFRAL, therapeutic strategies to precisely and effectively neutralize this axis have been limited. In this study, we demonstrate that de novo designed protein minibinders targeting the GDF15–GFRAL interface not only reverse cancer cachexia across multiple tumor models but also restore responsiveness to immune checkpoint blockade.

A key strength of this work lies in the use of structure‐guided de novo protein design to directly target the receptor‐binding surface of GDF15. By integrating RFdiffusion, ProteinMPNN, and AlphaFold‐based structure prediction, we generated compact minibinders that bind GDF15 with sub‐nanomolar affinity and high structural fidelity. Extensive mutagenesis and charge‐complementary rescue experiments provide compelling functional evidence that neutralization occurs through the intended, precisely engineered interface, establishing a direct causal link between design, binding mode, and biological activity. Although broader cross‐reactivity profiling was not performed in the current study, several lines of evidence support the specificity of the designed minibinder. Structural and mutational analyses demonstrated that binding depends on a defined set of GDF15 interface residues, as mutations at these positions markedly impaired minibinder recognition. Notably, these residues are poorly conserved among other members of the TGF‐β superfamily. GDF15 itself is one of the most sequence‐divergent TGF‐β family cytokines, sharing relatively low sequence similarity with canonical TGF‐β family members such as TGF‐β1, BMPs, and Activins. Together, these observations suggest that off‐target recognition of other TGF‐β family cytokines is structurally unlikely. These data highlight the power of modern computational protein design to generate antagonists with antibody‐like potency but substantially reduced molecular size and potentially favorable biophysical properties.

Functionally, GDF15 minibinders effectively suppressed downstream GDF15–GFRAL signaling, attenuated immediate early gene induction, and reversed hallmark features of cachexia, including progressive weight loss and reduced survival. Cancer cachexia is a multifactorial syndrome involving not only body‐weight loss but also skeletal muscle wasting, adipose tissue depletion, anorexia, and systemic metabolic alterations. In the study, additional assessment of muscle and adipose tissue mass further supports that GDF15 minibinder treatment alleviates cachexia‐associated tissue wasting. Nevertheless, more detailed metabolic profiling, including longitudinal food intake, energy expenditure, and circulating metabolic biomarkers, will be important in future studies to fully define the systemic physiological effects of GDF15 neutralization. Recent studies have also expanded the biological roles of GDF15 beyond cachexia and appetite regulation [34, 35]. In the tumor microenvironment, GDF15 has been implicated in inflammatory signaling, oxidative stress, neutrophil infiltration, tumor–stromal interactions, and immune suppression. These findings support the concept that GDF15 acts as a pleiotropic regulator of both systemic physiology and local tumor immunity, providing additional rationale for therapeutic blockade of this pathway.

Beyond cachexia, our findings establish a mechanistic link between GDF15 signaling and resistance to immune checkpoint blockade. Neutralization of GDF15 restored sensitivity to anti–PD‐1 therapy in an otherwise resistant tumor model, accompanied by increased CD8^+^ T cell infiltration and effector function. The complete abrogation of therapeutic benefit upon CD8 depletion demonstrates that the synergy between GDF15 neutralization and PD‐1 blockade is strictly dependent on cytotoxic T cell–mediated immunity. These findings are consistent with previous reports demonstrating that tumor‐derived GDF15 suppresses anti‐tumor immunity by impairing LFA‐1‐dependent T‐cell recruitment into the tumor microenvironment, thereby limiting responses to immune checkpoint blockade [14]. Notably, antibody‐mediated GDF15 inhibition has been shown to restore T‐cell infiltration and sensitize tumors to anti‐PD‐1 therapy. The comparable therapeutic phenotypes observed in our study further support that the de novo designed minibinder can effectively phenocopy established antibody‐based GDF15 blockade in vivo. These results support a model in which GDF15 promotes a systemic and local immunosuppressive state that limits effective antitumor immunity, and its neutralization reopens a therapeutic window for immunotherapy.

Despite the promising therapeutic efficacy observed in this study, several important considerations remain for future clinical translation. In particular, the potential immunogenicity of de novo designed protein therapeutics will require careful evaluation in long‐term preclinical and clinical studies. Although compact minibinders may offer advantages such as high structural stability, precise interface targeting, and reduced molecular complexity, immune responses against engineered proteins remain a potential challenge. Future optimization strategies, including sequence humanization, immunogenic epitope reduction, and pharmacokinetic engineering, may further improve the safety and translational potential of GDF15‐targeting minibinders. In addition, comprehensive long‐term toxicity, pharmacokinetic, and biodistribution studies will be necessary during future preclinical development.

What's more, although the MC38‐hGDF15 model provides a robust platform for investigating GDF15‐mediated cachexia and immunotherapy resistance, several limitations should be acknowledged. In particular, ectopic overexpression of human GDF15 may not fully recapitulate the dynamic regulation and heterogeneity of endogenous GDF15 expression observed in clinical tumors. Nevertheless, this engineered model enables controlled functional evaluation of GDF15 blockade in an immunocompetent setting and allows direct assessment of the therapeutic activity of human GDF15‐targeting minibinders in vivo. More broadly, engineered preclinical tumor models are valuable for testing defined therapeutic hypotheses, but their interpretation should consider the complexity of clinical tumor microenvironments and disease‐specific contexts, as also highlighted in recent studies of multimodal tumor immunotherapy models [36]. Future studies using additional endogenous or genetically engineered tumor models may further strengthen the translational relevance of these findings.

Together, this work positions the GDF15–GFRAL axis as a central node linking cancer cachexia, immune dysfunction, and therapeutic resistance. More broadly, it illustrates how de novo protein design can be leveraged to rapidly generate potent, mechanism‐defined biologics against challenging targets. Future studies will be required to optimize pharmacokinetics, assess long‐term safety, and explore clinical translation, but our findings establish a strong foundation for the development of next‐generation cachexia therapeutics with the potential to improve both quality of life and treatment outcomes in patients with cancer.

### 4.1 Declaration of Generative AI and AI‐Assisted Technologies in the Manuscript Preparation Process

During the preparation of this work, the author(s) used ChatGPT 5 (OpenAI) for language editing and manuscript organization. After using this tool, the author(s) reviewed and edited the content as needed and take full responsibility for the content of the published article.

## Author Contributions

**Haitao Wang** and **Tianzhen Hua** performed the majority of the experimental work, including protein expression and characterization, in vitro functional assays, and in vivo tumor and cachexia models. **Meiling Wang** and **Bingbing Meng** conducted the computational design and screen. **Yue Zhang** contributed to protein design and optimization. **Chengxu Jiang** provided assistance with animal experiments. **Hongqi Yang** supervised the immunological experiments, guided cachexia characterization and in vivo phenotypic analyses, and led the manuscript revision process. **Yunhe Gao** and **Jian Bo** conceived the study, supervised the research. All authors analyzed the data, discussed the results, and contributed to the writing of the manuscript.

## Funding

This study was supported by Major Logistics Project of the Chinese People's Liberation Army (Grant Nos. AWS21J003, ALB23J008); Innovation Transcend Project of National Defense Science and Technology (Grant No. 221‐CXCY‐N101‐09‐11); Key Logistics Project of the Chinese People's Liberation Army (Grant No. 145BHQ090003000×11‐3‐1‐1); Beijing Natural Science Foundation (L254098); The Postdoctoral Fellowship Program of CPSF (Grant No. GZC20242288).

## Conflicts of Interest

The authors declare no conflicts of interest.

## Data Availability

The data that support the findings of this study are available on request from the corresponding author. The data are not publicly available due to privacy or ethical restrictions.
